# The Pulte Quarterly

**Published:** 1890-06

**Authors:** 


THE PULTE QUARTERLY. Published at 124 West Seventh Street, Cincinnati, Ohio. April, 1890.

This is a new journal, the first number of which (for April) is before us. It is devoted to the interests of Pulte Medical College, of Cincinnati, Ohio.

The editor is Thomas M. Stewart, M. D. Associate editors are J. D. Buck, M. D., Phil. Porter, M. D., C. E. Walton, M. D., G. C. McDermott, M. D., and C. D. Crank, M. D. The new journal contains the news of Pulte College and has besides an assortment of clinical matter. W. M. J.

